# 4.J. Workshop: Health system performance assessment in Europe – recent developments, evidence, and challenges

**DOI:** 10.1093/eurpub/ckac129.243

**Published:** 2022-10-25

**Authors:** Organised by: Berlin University of Technology, Chair persons: Reinhard Busse (Germany), Carine Van de Voorde (Belgium)

## Abstract

Assessing the performance of health systems is essential to initiate policy processes that further strengthen health systems to improve population health. Starting with WHO's World Health Report of 2000, Health System Performance Assessments (HSPA) gained increasing attention in research and policy. By now, several comparative HSPA initiatives of international organisations (e.g., OECD's Health at a Glance) and country specific HSPA (e.g., Sweden, Netherlands, Austria, Belgium) were implemented in Europe. Further countries are currently in the process of developing or piloting an HSPA (e.g., Germany). The objective of the workshop is to foster and strengthen the interchange of different HSPA initiatives in Europe and beyond. While each health system has its specifics, initiatives likely face similar challenges in the implementation and ongoing development, which holds a lot of potential for learning from each other. The workshop is supposed to be a platform for the exchange of and about HSPA initiatives and related topics by bringing together stakeholders of different countries and professions. Participants are supposed to gain new insights into recent activities in Europe and will be able to discuss challenges and lessons learned. The presentations give insights into recent activities of selected HSPA initiatives in Europe. One presentation originates from the Belgian HSPA which is well established. This presentation will focus on the transversal dimension of equity, on which a separate report was recently published. The second presentation is about the German HSPA which is still in development and currently tested in a pilot study. Both presentations represent HSPA initiatives at different stages, which provides a good basis for the following panel and discussion with all workshop participants. The two presentations of about 20 minutes each will be combined with a panel discussion. Afterwards, there will be room for exchange and discussions between all participants and presenters.

**Key messages:**

• Recent developments of HSPA initiatives in Europe will be presented along two examples from Belgium and Germany and lessons learned and challenges will be discussed.

• The workshop provides a platform for the exchange of and about HSPA initiatives and related topics by bringing together stakeholders of different countries and professions.

